# Skipping Breakfast Is Associated with an Atherogenic Lipid Profile in Overweight and Obese Prepubertal Children

**DOI:** 10.1155/2020/1849274

**Published:** 2020-10-10

**Authors:** A. Blasetti, S. Franchini, V. Castorani, L. Comegna, E. Fornari, F. Daniele, G. Prezioso, C. Piona, V. Federico, D. Zona, I. Bresadola, F. Chiarelli, C. Maffeis

**Affiliations:** ^1^Department of Pediatrics, “G. d'Annunzio” University-Chieti, Pescara, Italy; ^2^Pediatric Diabetes and Metabolic Disorders Unit, University of Verona, Verona, Italy; ^3^Department of Medical, Oral and Biotechnological Sciences, “G. d'Annunzio” University-Chieti, Pescara, Italy; ^4^^“^SS Annunziata” University Hospital, Unit of Clinical Molecular Biology, “G. d'Annunzio” University-Chieti, Pescara, Italy

## Abstract

**Background:**

Skipping breakfast has been associated with a higher risk of obesity and cardiovascular (CV) risk factors. However, it is not known if skipping breakfast is also correlated with CV risk factors independently from obesity. The mechanisms explaining the role of skipping breakfast on promoting fat accumulation as well as CV risk are not known. Hormones, in particular, insulin-like growth factor-1 (IGF-1), may potentially play a role in the metabolic profile of breakfast skippers.

**Aim:**

This cross-sectional study aims to test, in a sample of overweight/obese children, the hypotheses that skipping breakfast is associated with a worse metabolic profile and that IGF-1 levels are associated with this unfavorable metabolic profile.

**Methods and Results:**

We enrolled 112 overweight/obese prepubertal children (3–12 years). Anthropometric characteristics (height SDS, weight SDS, and body mass index (BMI) *z*-score) were measured. Blood samples were collected to evaluate glucose and lipid metabolisms and hormone profile (growth hormone (GH), IGF-1, insulin, and cortisol). The triglycerides/high-density lipoprotein (HDL) cholesterol ratio was calculated as a predictor of cardiovascular risk. Children were divided into two groups according to breakfast habits: consumers (≥5 weekly; *N* = 76) and skippers (≤4 weekly; *N* = 36). Glycaemia, total and low-density lipoprotein (LDL) cholesterol, triglycerides (*p* < 0.05), and triglycerides/HDL cholesterol ratio (*p* < 0.001) were higher, while HDL cholesterol was lower (*p* < 0.01) in skippers as compared to consumers. IGF-1 concentrations were inversely correlated with LDL cholesterol (*r* = −0.279, *p*=0.013) and directly correlated with HDL cholesterol (*r* = 0.226, *p*=0.047). IGF-1 correlated positively with HDL cholesterol (*r* = 0.266, *p*=0.045) in consumers and correlated negatively with LDL cholesterol (*r* = −0.442, *p*=0.024) in skippers. Breakfast consumption among prepubertal overweight/obese children showed a better lipid profile in comparison with those who skipped breakfast [OR: 0.165 (95% CI: 0.053–0.518), *p*=0.001]; these latter odds of the increased triglycerides/HDL cholesterol ratio was 6.1-fold higher.

**Conclusions:**

Breakfast skippers show a worse lipid profile when compared to breakfast consumers. IGF-1 might play a role as an independent modulator of lipid metabolism.

## 1. Introduction

Obesity is the most common metabolic disorder worldwide [1] and is associated with cardiovascular risk factors such as hyperlipidemia, hyperglycemia, and hypertension even in children and adolescents [2]. Breakfast habits have been suggested to have direct effects on the development of obesity as well as on the metabolic profile and subsequent cardiometabolic risk [3]. Therefore, it is reasonable to expect that food-intake dysregulation associated with a chronic breakfast skipping behavior may promote excessive body fat accumulation and metabolic derangement, whereas regular breakfast consumption has been associated with a healthier cardiovascular status due to a better lipid profile and lower adiposity [4, 5]. An interesting association between skipping breakfast and obesity has been reported in both children and adolescents [6, 7]. Moreover, the mechanisms leading to extra fat accumulation in breakfast skippers are unknown although the overconsumption of high-energy-dense food in the morning, aimed at satisfying an increased appetite, has been reported as a reasonable contributing factor to body fat gain [6, 7].

Usually, obese children and adolescents have high levels of circulating triglycerides (TGs) and low levels of high-density lipoprotein (HDL) cholesterol [8]. Both factors have been associated with increased cardiovascular risk and insulin resistance [2, 8, 9]. Indeed, the triglyceride-to-HDL cholesterol (TG/HDL ch.) ratio has been proposed as a practical and useful index for predicting cardiovascular risk [10, 11]. Although, a clear association between lipid profile and cardiovascular risk has been demonstrated in obese children and adolescents [11], a relation between breakfast consumption and lipid profile, regardless of the level of overweight, has not been explored.

Hormones are potential contributors to the metabolic consequences of skipping breakfast, in particular, the growth hormone/insulin-like growth factor-1 (GH/IGF-1) axis. An association between IGF-1 and cardiovascular diseases has been reported [12], as well as an inverse association between IGF-1 and lipid profile [13]. In a recent review, Hawkes and Grimberg underlined that IGF-1 concentrations in obese individuals were similar to, or higher than, nonobese peers, despite a reduction in circulating GH levels and a short-term caloric restriction were associated with IGF-1 reduction in obese children [14]. In particular, total IGF-1 concentrations did not seem to be significantly increased in obesity, whilst the amount of free IGF-1 relative to total IGF-1 was increased [14]. Inzaghi et al. highlighted that IGF-1 was directly correlated with HDL cholesterol in children under 10 years, supporting the hypothesis that IGF-1 may be considered as an early biomarker of cardiovascular risk [15].

The aim of the present study was to test the hypothesis that skipping breakfast entails a bad metabolic profile. The relation between breakfast consumption and metabolic and endocrine fasting profiles and the role of IGF-1 in this association were investigated in a population of overweight and obese prepubertal children.

## 2. Materials and Methods

The study population included 112 Caucasian prepubertal children divided into two groups based on breakfast habits: skippers (*n* = 36) and consumers (*n* = 76). Children were recruited between August 2018 and March 2019 at the Department of Pediatrics, University of Chieti, Chieti, Italy, and at the Outpatient Obesity Clinic, Pediatric Diabetology and Metabolic Disorders Unit, University of Verona, Verona, Italy. Children were admitted in the study only after complete recovery from the minor diseases they had been admitted and the discharge from the hospital.

Inclusion criteria were as follows: age (3 to 12 years), European ancestry, and a diagnosis of overweight or obesity, according to the reference values of body mass index (BMI > 85^th^ percentile for age and gender) for children and adolescents [16]. A complete availability of anthropometric measurements, laboratory tests, medical and family history, and breakfast habits were collected. Exclusion criteria were as follows: clinical signs of puberty (Tanner stage > 1), drugs affecting body composition, lipid and endocrine profiles, secondary forms of obesity and chronic diseases, and a diagnosis of feeding and eating disorders.

Two different groups were identified on the basis of their breakfast habits: skippers (*n* = 36), i.e., children having breakfast less than 4 days per week, and consumers, i.e., children having breakfast for at least 5 days per week (*n* = 76). Breakfast was defined as the consumption of at least one meal between 05 am and 10 am [17]. Even though self-reporting breakfast consumption/skipping to gauge breakfast habits could expose potential biases, it was chosen supported by available data that suggest that accuracy in self-reporting breakfast consumption or not is more than acceptable also in children [7, 18]. Breakfast frequency was assessed by a questionnaire administered during enrollment, with the query: “during the past week, how many days did you have breakfast?” Responses included never to 4 days (skippers) and 5 days to everyday (consumers). We did not collect information about socioeconomic statuses, physical activity, and nutrient and food type intake. The study was performed in accordance with the ethical standards laid down in the 1964 Declaration of Helsinki. Written informed consent was obtained from the prepubertal children who participated in the study and their parents.

### 2.1. Physical Characteristics

Physical examination included pubertal development assessment (Tanner's stage) and anthropometry (weight and height). Body weight was taken with a digital scale to the nearest ±0.1 kg. Height was measured in triplicate with a wall-mounted Harpenden stadiometer to the nearest ±0.1 cm. BMI was used as the fatness index in all children, and the BMI *z*-score for age and sex was also calculated using the reference data for Italian population [12]. Prepubertal children were defined as Tanner's stage <2 for both sexes (absence of breast bud in females and testicle size <4 ml in males) [19].

### 2.2. Laboratory Procedures

Venous blood samples were collected from the antecubital vein after an overnight fast. Plasma glucose was measured by using the glucose oxidase method. TGs, total cholesterol, and HDL cholesterol were measured with an enzymatic-calorimetric test. Low-density lipoprotein (LDL) cholesterol was inferred by using the Friedewald formula [20]. TG/HDL ch. ratio was calculated, and a cutoff value of TG/HDL ch. ratio >2.27 was chosen as the predictor of higher risk of insulin resistance [21]. Serum IGF-1 was quantified by the ELISA Sandwich enzyme immunoassay (LIAISON® BRAHMS PCT® II GEN). GH and insulin were measured with dual antibody sandwich-type immunoassays (2000 Automated Immunoassay Analyzer). Insulin sensitivity was estimated using the homeostasis model assessment index (HOMA-IR), with the following formula: fasting serum insulin (U/mL) × fasting glucose (mmol/L)/22.5. Cortisol was determined using colorimetric detection by a Cortisol ELISA kit.

### 2.3. Data Analyses

Data input and basic evaluation were carried out using SPSS version 19 software for Windows (SPSS, Chicago, IL). Normally distributed values were expressed as mean ± standard deviation. *χ*^2^ test was used to compare proportions. The Mann–Whitney test was used for comparison between groups. Pearson's correlation analysis was carried out to measure correlations between variables. A binary logistic step-down regression analysis was run using the TG/HDL ch. ratio as a dependent variable (0 ≤ 2.27, 1 > 2.27) and age, gender, BMI *z*-score, IGF-1, HOMA-IR, and breakfast consumption (0 = consumers; 1 = skippers) as independent ones. Variation of lipid parameters according to IGF-1 was analyzed by general linear models adjusted for age, gender, and BMI z-score. A *p* value <0.05 was considered statistically significant. Finally, power analysis was done considering a study design with two independent groups and a continuous variable. A total sample size of 99 prepubertal children was obtained, considering an enrollment ratio 1 : 2, *α* = 0.05, and power of 80%.

## 3. Results

### 3.1. Physical and Biochemical Characteristics

The anthropometric and biochemical characteristics of the study population are summarized in Table 1. There were no significant differences between the two groups (skippers vs. consumers) for age, anthropometric characteristics, GH, IGF-1, and cortisol. Instead, plasma glucose, total cholesterol, LDL cholesterol, and TGs were significantly higher (*p* < 0.05), and HDL cholesterol was significantly lower (*p* < 0.01) among skippers as compared to consumers. Moreover, the TG/HDL ch. ratio was significantly higher (*p* < 0.001) in skippers as compared to consumers.

### 3.2. Correlation Analyses

After adjustment for age, gender, and BMI *z*-score, IGF-1 resulted in being inversely and significantly correlated with LDL cholesterol (*r* = −0.279, *p*=0.013) and directly correlated with HDL cholesterol (*r* = 0.226, *p*=0.047). IGF-1 did not correlate with total cholesterol, triglycerides, and TG/HDL ch. ratio (*r* = 0.174, *p*=0.128; *r* = −0.005, *p*=0.967; *r* = −0.190, *p*=0.085, respectively).

**Figure 1:**
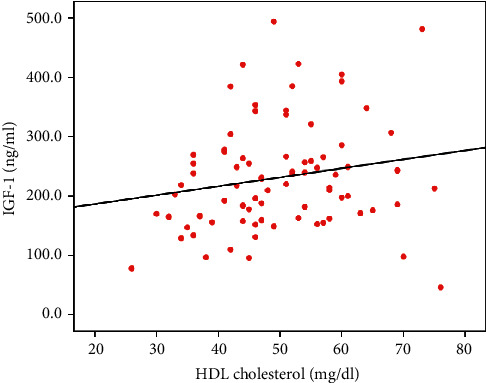
Linear regression plot of IGF-1 and HDL cholesterol among breakfast consumers. A positive statistically significant correlation was found between IGF-1 and HDL cholesterol (*r*=0.266, *p*=0.045). IGF-1: insulin-like growth factor-1; HDL: high-density lipoproteins.

**Figure 2:**
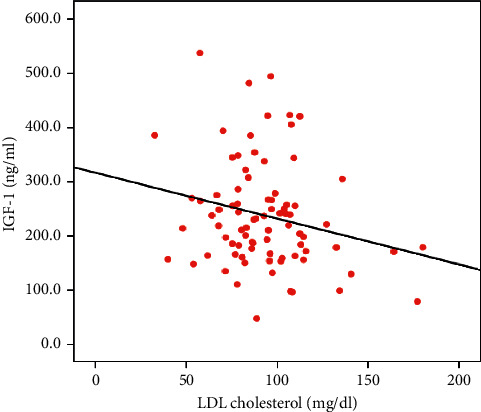
Linear regression plot of IGF-1 with total and LDL cholesterol among breakfast skippers. A negative statistically significant correlation was found between IGF-1 and LDL cholesterol (*r*=−0.442, *p*=0.024). IGF-1: insulin-like growth factor-1; LDL: low-density lipoproteins.

A direct correlation was found between IGF-1 and HDL cholesterol (*r* = 0.266, *p*=0.045) in consumers (Figure 1) and an inverse correlation between IGF-1 and LDL cholesterol (*r*=−0.442, *p*=0.024) in skippers (Figure 2). No statistically significant associations were found among other markers of endocrine and metabolic profiles (data not shown).

Binary logistic regression model is shown in Table 2. In prepubertal overweight and obese children, breakfast consumption was associated with a better lipid profile as compared to breakfast skipping [OR: 0.165 (95% CI: 0.053–0.518), *p*=0.001]; these latter odds of the increased triglycerides/HDL ch. ratio were 6.1-fold higher.

## 4. Discussion

The main finding of this study is that skipping breakfast is associated with a worse metabolic profile and a higher cardiovascular risk as compared to almost daily or daily breakfast consumption. An association between IGF-1 serum concentrations and specific markers of the lipid profile was found, suggesting a potential effect on the cardiovascular status.

Unfortunately, skipping breakfast is becoming common in Western countries, and it is associated with increasing incidence of overweight and obesity. Currently, frequency, content, and regularity of breakfast consumption are crucial issues among children and adolescents [3, 22]. In our study population, severity of overweight and obesity (defined by the BMI z-score) did not differ between skippers and consumers. The comparable level of overweight and obesity among skippers and consumers offers the chance to explore the effect of breakfast consumption on cardiovascular risk factors, regardless of the effects of the BMI *z*-score.

Freitas et al., investigating the association between skipping breakfast and glucose and lipid profiles in a population of 174 obese children and adolescents, reported that eating breakfast was inversely related to glycaemia, triglycerides, and very-low-density lipoprotein (VLDL) cholesterol, even after adjustment for age, sex, abdominal adiposity, and parents' education status, without differences in BMI [23]. Accordingly, we found significantly higher fasting glucose, triglycerides, total and LDL cholesterol, and lower HDL cholesterol in skippers as compared to consumers. Moreover, increased fasting glycaemia among skippers in our series confirms the data reported by Donin et al. in the Child Heart and Health Study in England (CHASE), reporting increased mean fasting glycaemia among skippers as compared to children having breakfast daily [18]. The worst metabolic profile found in skippers is in agreement with the results of the HELENA Study, which analyzed breakfast habits among a large sample of European adolescents. In this study, a significantly higher BMI, fasting insulin, and HOMA-IR values, in skippers versus consumers, as well as a worse lipid profile (lower total cholesterol and LDL cholesterol), were shown [5]. Similarly, in a prospective analysis of breakfast habits in a cohort of 2216 adolescents, Timlin et al. reported that breakfast frequency was inversely associated with weight gain and BMI changes in a dose-response association [24].

The possible reasons explaining the role of skipping breakfast in modifying the lipid profile in overweight and obese youth are not clear. It has been suggested that macro- and micronutrient supplies with breakfast may affect the dietary behavior during the entire day, promoting adherence to a healthier diet, which is associated with a better metabolic condition [25]. A positive impact of regular meals and intermeal intervals on body composition and metabolic profile has been demonstrated [4, 22]. Skipping breakfast may increase morning hunger, facilitating weight and fat mass increase due to high intake of snacks and high-calorie foods [26].

We found a higher TG/HDL ch. ratio in breakfast skippers as compared to consumers. TG/HDL ch. ratio has been suggested as a predictor of cardiovascular risk and insulin resistance in adults, children, and adolescents [9]. de Giorgis et al. investigated the association among TG/HDL ch. ratio and other cardiovascular risk factors and early signs of vascular damage in prepubertal children. They highlighted a higher ratio between obese children as compared with thin controls and a significant association with the carotid intima-media thickness and a greater intima-media thickness and insulin resistance among children in the upper tertile of the TG/HDL ch. ratio [10]. Moreover, data from the Third National Health and Nutrition Examination Survey (NHANES III) underlined that high triglycerides and low HDL cholesterol levels are the most common laboratory alterations in the metabolic syndrome involving adolescents [8]. These data were also confirmed in an Italian independent cohort of children and adolescents [2]. In our study, breakfast habits were associated with the TG/HDL ch. ratio, independently of age, sex, and overweight/obesity severity. As shown in Table 2, overweight and severity of obesity do not seem to affect the TG/HDL ch. ratio (*p*=0.058). It must be noted that our study population consists exclusively of overweight and obese prepubertal children, so there are no statistically significant differences between the 2 groups (skippers vs. consumers). Homogeneity of the BMI *z*-score may strengthen the result of an influence of breakfast habits on the TG/HDL ch. ratio. This finding supports the hypothesis that breakfast habits may affect the metabolic risk profile of overweight and obese children. Hence, regular breakfast consumption in prepubertal children seems to guarantee a healthier and more protective lipid profile as compared to breakfast skipping, regardless of the obesity level. This result has potential clinical and public health implications in the prevention of cardiovascular disorders. However, future studies with larger populations are needed to better define whether the severity of obesity may influence the lipid profile according to breakfast habits, by measuring waist circumference as a marker of fat mass distribution.

Lipid profile is modulated by a complex network of biochemical processes, and the physiological mechanisms are not completely understood. In these processes, a potential role for IGF-1 has been proposed, owing to the well-known association between IGF-1 levels and lipid profile although conflicting results have been reported [17]. In our study, we found an inverse correlation between IGF-1 levels and LDL cholesterol, as opposed to a direct association with HDL cholesterol. In each group, we found a direct correlation between IGF-1 and HDL cholesterol among consumers and an inverse correlation between IGF-1 and LDL cholesterol among skippers. Succurro et al. detected a direct correlation of IGF-1 with HDL cholesterol in 1.004 overweight/obese adults, concluding that IGF-1 may be an independent modulator for HDL cholesterol levels [27]. To date, the physiological pathway through which IGF-1 may modify HDL metabolism has not been clearly demonstrated. High IGF-1 levels could increase insulin sensitivity, while insulin resistance has been associated with lower IGF-1 and enhanced metabolism of HDL particles by hepatic lipase [28]. Furthermore, IGF-1 downregulates the expression of class BI hepatic scavenger receptors that mediate HDL cholesterol uptake into hepatocytes. It is speculative that a reduction in circulating IGF-1 levels may upregulate HDL cholesterol uptake by hepatic SR-BI with a resulting decrease in serum HDL cholesterol levels [29]. The mechanisms reported above involving the interdependence of HDL and LDL cholesterol metabolism may also potentially justify why children skipping breakfast showed an inverse association between IGF-1 and LDL cholesterol.

Interestingly, Eggert et al. also proposed that IGF-1 might represent a risk marker than a risk factor for alterations in lipid metabolism [30].

The present study highlights the potential cofactors leading to the expression of a higher cardiometabolic risk phenotype associated with skipping breakfast. The relatively young age of the sample of overweight and obese prepubertal children seems adequate to exclude the presence of obesity-related comorbidities that may have contributed to confound the analysis. Similarly, the prepubertal stage chosen in the present study is an important factor that allows bypassing of puberty-related metabolic and hormonal changes. We are aware of some limitations of the study: a relatively small sample size, as well as not having considered other relevant variables.

In conclusion, skipping breakfast was associated with a cardiometabolic risk profile in overweight and obese prepubertal children and suggested a role for IGF-1 as a potential modulator of lipid metabolism. Further studies including larger samples of multiethnic children that consider variables such as dietary intake, physical activity, and socioeconomic statuses should be undertaken, applying experimental designs that do not limit the possibility of proving a cause-effect relation between breakfast habits and cardiometabolic risk, especially regarding the effect of having breakfast on the relationship between IGF-1 and LDL and HDL cholesterol.

## Figures and Tables

**Table 1:** General characteristics of the study population^*∗*^: skippers vs. consumers.

Characteristics	All	Skippers	Consumers	*p*
*N*	112	36	76	—
Male/female	42/70	14/22	28/48	0.836
Age (years)	8.9 ± 1.8	8.8 ± 2.3	9.0 ± 1.5	0.630
Height SDS	0.67 ± 1.06	0.62 ± 1.12	0.70 ± 1.04	0.727
Weight SDS	1.66 ± 0.76	1.76 ± 0.82	1.61 ± 0.73	0.328
BMI *z*-score	1.71 ± 0.55	1.79 ± 0.66	1.66 ± 0.49	0.241
Plasma glucose (mg/dl)	88.5 ± 8.1	93.3 ± 8.7	86.2 ± 6.7	**<0.001**
Insulin (*μ*U/ml)	10.8 ± 5.1	10.8 ± 5.2	10.8 ± 5.1	0.943
HOMA-IR	2.4 ± 1.2	2.5 ± 1.3	2.3 ± 1.1	0.417
Total cholesterol (mg/dl)	157.9 ± 26.5	166.2 ± 30.5	153.6 ± 23.4	**0.037**
HDL cholesterol (mg/dl)	50.3 ± 11.4	45.4 ± 12.1	52.7 ± 10.3	**0.005**
LDL cholesterol (mg/dl)	93.1 ± 26.1	104.5 ± 32.3	87.3 ± 20.3	**0.003**
Triglycerides (mg/dl)	75.5 ± 31.8	90.4 ± 39.7	68.2 ± 24.3	**0.002**
TG/HDL ch. ratio	1.66 ± 1.02	2.30 ± 1.39	1.37 ± 0.64	**<0.001**
GH (ng/ml)	0.59 ± 1.27	0.81 ± 1.73	0.48 ± 0.99	0.201
IGF-1 (ng/ml)	235.0 ± 91.7	224.5 ± 99.4	240.0 ± 91.1	0.405
Cortisol (ng/dl)	11.2 ± 4.3	11.5 ± 4.8	11.1 ± 4.0	0.652

^*∗*^Data are expressed as mean ± standard deviation (normally distributed). BMI: body mass index; HOMA-IR: homeostatic model assessment for insulin resistance; HDL: high-density lipoproteins; LDL: low-density lipoproteins; TG: triglyceride; GH: growth hormone; IGF-1: insulin-like growth factor-1.

**Table 2:** Binary logistic regression model.

Variables	TG/HDL cholesterol ratio		
	*β*	*p*	OR
Sex (male = 1; female = 2)	0.162	0.176	2.292
Age (years)	0.077	0.753	0.943
HOMA-IR	0.748	0.911	1.032
IGF-1 (ng/ml)	0.023	0.181	1.004
BMI *z*-score	0.323	0.058	3.655
Breakfast habits (skippers = 1, consumers = 2)	−0.421	0.001	0.165

Dependent variable: TG/HDL ch. ratio. HOMA-IR: homeostatic model assessment for insulin resistance; HDL: high-density lipoproteins; TG: triglyceride; IGF-1: insulin-like growth factor-1; BMI: body mass index.

## Data Availability

The datasets generated and/or analyzed during the current study are available from the corresponding author on reasonable request.
